# General Surgery: Requirements, Rationale, and Robust Results

**DOI:** 10.1055/s-0042-1758659

**Published:** 2022-12-02

**Authors:** Sunil Jain, Ashok Puranik

**Affiliations:** 1 Department of Paediatrics, Military Hospital Secunderabad, Secunderabad, Telangana, India; 2 Trauma Care and Acute Care Surgery, Monash University Australia, Melbourne, Australia; 3 Executive Director, All India Institute of Medical Sciences (AIIMS) Guwahati, Assam, India

**Keywords:** surgical care, emergency, competency, case-fatality rates, public health, technology, minimally invasive surgery, telementoring, professionalism, virtual surgical planning, curriculum

## Abstract

Safe, timely, and affordable surgical care is desirable worldwide, but is largely an unmet need. Surgical care is recognized as an important component of public health. Vision for sustainable surgical development is desirable, and general surgeons can contribute substantially toward this mission.

In the absence of surgical care, case-fatality rates are high for common and easily treatable conditions. These include congenital anomalies, hernia, fractures, appendicitis, etc. Solution is surgical care. Results of surgery on time are rewarding.

General surgeons, as per the Medical Council of India, are required to (1) recognize the health needs of the community and carry out professional obligations, (2) be competent, and (3) be aware of the contemporary advances and developments in the discipline concerned. All this ensures that the general surgeon should be able to treat almost all surgical conditions effectively.

With timely, cautious, careful, and tactful surgeries, general surgeons should be able to deliver robust results both electively and in emergency. All this in the true spirit of “Vayam Sevaamahe – We are for service” the motto of the Association of Surgeons of India. General surgeons should boost the best what was termed “ *Professional patriotism* ” in the historic Flexner report.

Surgical care is recognized as an important component of public health. 1 Five billion people worldwide do not have access to safe, timely, and affordable surgical and anesthesia care. This results in over 18 million deaths each year and one-third of the global burden of disease. 2

Against this background, our article's aim is to (1) analyze general surgery (GS) requirements, (2) build upon the rationale for the role general surgeons should be playing, and (3) catalyze the results which general surgeons can produce.

Strategies for sustainable surgical development are desirable and should aim for quality dividends quantitatively. Access to the full spectrum of health care, including surgical care by all has to be achieved. Surgically treatable conditions account for a significant proportion of the total global burden of disease, and surveys of need have shown that it is largely unmet. 3 4 Recently, it has been reported that the world's population without access to surgical care is significantly greater than previously reported. The modeling study estimates that at least 4.8 billion people do not have access to surgical care. 5 General surgeons can play an important role. Personal aptitude for surgery and professionally armed with skills, general surgeons can deliver results.

Professional approach to health for all and advancing modern surgery, demands wide expertise by all against the present trend of limited expertise by only a few for a limited field—the so-called sub(super)-specialization. Majority of the surgical conditions can be dealt by general surgeons and they are trained in it. Holistic healing requires leadership, understanding of human as a whole, and not just organ- or system-specific superspecialist surgeons.

## The Requirements

Lancet Commission on Global Surgery estimated need of 5,000 surgeries per year per 100,000 population. 6 In India, it has been estimated that 3,646 surgeries are required per 100,000 Indian population per year. 7 Of these, 26% would be general surgical procedures, 20% would be obstetrics and gynecological surgeries, and 13% would be ophthalmological surgeries.

In the absence of surgical care, case-fatality rates are high for common and easily treatable conditions including congenital anomalies, hernia, fractures, appendicitis, etc. These conditions are ubiquitous. Solution is surgical care. Results of surgery on time are rewarding. 8

India's doctor-population ratio of 1:854 is better than the World Health Organization (WHO) standard of 1:1,000. 9 Estimates of the stock of surgeons in India range between 1.5 and 6.8 per 100,000 population. India continues to experience shortages of health workers despite impressive increases in production capacity in recent years. The production capacity of surgical specialists in India has experienced an upward trend in recent years, and in 2015 it was estimated to be 2,131. 10 The number of super- or subspecialist surgeons are abysmally low. General surgeons have to and can play an important role.

With universal coverage of essential surgery in low-income and middle-income countries, 1.5 million deaths per year could be averted, representing 6.5% of all avertable deaths in these countries. 11 Morbidity figures can be much more. It has been suggested that as an interim measure, multiskill training of general doctors to reduce the deficit of surgical specialists, particularly at community health centers, should be expanded. 10

Worldwide statistics reveal other aspects as well. Available statistics show that over 44% of WHO Member States report to have less than 1 physician per 1,000 population. Health workers are distributed unevenly across the globe. Countries with the lowest relative need have the highest numbers of health workers, while those with the greatest burden of disease must make do with a much smaller health workforce. 12

It has been commented that essential surgery provisioning in rural and remote areas can be only partly met both in developed and in low- and middle-income countries. It may take years to solve the problem of unmet needs. 13 General surgeons should play an increasingly important role in reaching and serving all.

The scenario of surgeons in the Indian Armed Forces is peculiar. The needs are both peace-time and war-time. In addition, surgeons of the Indian Armed Forces play an important role in relief operations. They also play important role by way of surgical camps like those of Op Sadbhavana, in remote and underserved locations.

## The Rationale

The World Federation for Medical Education Global Standards 2015 for Postgraduate Medical Education stipulates to base the mission on consideration of the health needs of the community or society. 14 Similarly, the General Medical Council, U.K., specifies that postgraduate training should be responsive to adapt to current and emerging patient and population needs. 15 All this, against the backdrop of requirements, points to an increasingly important role the general surgeons should be playing in serving the surgical needs of populations.

The goal of postgraduate medical education shall be to produce competent specialists. General surgeons awarded MS degrees are expected to have the following competency as per the Medical Council of India (now the National Medical Commission) 16 :

First, recognize the health needs of the community, and carry out professional obligations ethically and in keeping with the objectives of the national health policy. Important implication is to meet all the surgical needs of the community.

Second, mastered most of the competencies, pertaining to the speciality, that are required to be practiced at the secondary and the tertiary levels of the health care delivery system. All the needs of communities, states, and nations can be met for by general surgeons in these levels of health care.

Third, be aware of the contemporary advances and developments in the discipline concerned. Surgeons have the enthusiasm, and with sustained efforts for ongoing training the general surgeon should be able to treat almost all surgical conditions effectively.

A meeting of experts for workable solutions for GS workforce shortfalls recommended to enhance the number of GS trainees and the breadth of training. It also recommended that minimally invasive surgery should largely return to GS. 17 Feasibility of minimally invasive surgical procedures in the outpatient setting is a further boost. 18 Similarly, an Indian peer review of teaching, training, and evaluation in GS has pointed out that it should be broad based and skills should be open and endoscopic. 19 General surgeons need to learn, practice, and master the latest trends. All this will lead to professional progress, self-satisfaction, and satisfaction of the masses.

Surgeons have long been considered leaders in adapting and improving their practice. 20 Latest evidence is useful and needs to be implemented. Engagement in a growing number of free-to-use “Web portals” (e.g., General Surgery—Medscape, available at http://www.medscape.com/generalsurgery/ ) and medical media communication (e.g., General Surgery News, available at http://www.generalsurgerynews.com/ ) is useful in assimilating the newest evidence.

Formal online courses, webinars, and remote telementoring all provide examples of surgical leadership. 21 All these should be used by general surgeons for progressive improvements.

Professionalization of modern surgery demands wide expertise by all against the present trend of limited expertise by only a few for a limited field—the so-called subspecialization.

Professionalism has been defined as “a set of values, behaviors, and relationships that underpins the trust the public has in doctors.” 22 Competence is an important attribute of professionalism, in relation to surgical practice. 23 Thus, a general surgeon should be competent in dealing with almost all conditions. Subspecialization expertise is required for only few conditions. Further, tactful supportive follow-up strategies can ensure healthy well-being after complex surgeries. 24 General surgeons can play an important role in this respect as well.

General surgeons need to be energized for excellence: It requires sustained energy for excellence, to achieve the targets, and to stay high on the results. With all this we should be able to meet the unmet needs. 25

## Robust Results

Improvements in antisepsis, advanced anesthetic techniques, and sophisticated surgical skills have led to improvement in outcomes for surgical patients. Surgery can be elective or performed in emergency. Identifying predictors of mortality and surgical complications has led to outcome improvements for a variety of surgical conditions. 26 Recent studies have shown that emergency GS patients are at uniquely higher risk for medical errors and complications following surgery, with emergency GS patients up to eight times more likely to die compared to patients undergoing the same procedure electively. The excess morbidity and mortality of emergency GS are not fully explained by preoperative risk factors, making emergency GS an excellent target for quality improvement projects. 27 28 All this points to need for timely and careful surgical intervention.

An illustrative example of need for timely surgical intervention is a common condition—inguinal hernia. A hernia not operated is a cause of morbidity, and complications of hernia are a potential cause of mortality. Risks of surgery are substantially less and results rewarding. Approximately 50% of inguinal hernias manifest clinically in the first year of life, most in the first 6 months. The incidence of complications associated with *elective* hernia repair (intestinal injury, testicular atrophy, recurrent hernia, wound infection) are low (≈1%), but rise to as high as 18 to 20% when repair is performed at the time of incarceration. Elective inguinal hernia repair can be safely performed in an outpatient setting with an expectation for full recovery within 48 hours. 29 All these point toward need of delivery by general surgeons.

Adverse events in surgery are an important problem globally. Many are preventable. The WHO Surgical Safety Checklist has been shown to reduce surgical complications and improve communication and teamwork in the operating theatre. 30 31 Feasible measures, such as WHO's Surgical Safety Checklist, lead to improvements in safety and quality. The basic and essential objectives for safe surgery according to WHO are: Operate on the correct patient at the correct site; Use method known to prevent harm from anesthetic administration, while protecting the patient from pain; Recognize and effectively prepare for life-threatening loss of airway or respiratory function; Recognize and effectively prepare for risk of high blood loss; Avoid inducing any allergic or adverse drug reaction known to be a significant risk for the patient; Consistently use method known to minimize risk of surgical site infection; Prevent inadvertent retention of instruments or sponges in surgical wounds; Secure and accurately identify all surgical specimens; Effectively communicate and exchange critical patient information for the safe conduct of the operation; Establish routine surveillance of surgical capacity, volume, and results. 32

An important quality improvement initiative is clinical audit. The process involves comparing aspects of care (structure, process, and outcome) against explicit criteria and defined standards. General surgeons should analyze results and compare performance against agreed standards with regular audit. It also documents track of personal clinical results. Rigorous evaluation of even the most simple techniques and conditions should be done. It can help to keep a surgeon stimulated throughout a long career and ensure good outcomes for patients, with cost benefits to the provider and a benefit to society as a whole. 33

With timely, cautious, careful, and tactful surgeries general surgeons should be able to deliver robust results both electively and in emergency. More sophisticated surgical operations with better results are increasingly performed. General surgeons should also continue to increase their skills and sophistication.

Advancements in technology hold great promise for the advancement of patient care. Modern technology enriches (1) proper assessment, (2) pertinent management, including surgical intervention, (3) progress monitoring, including for adverse events, 34 (4) prevention of progress/worsening/complications, (5) professional standards achievement, improvement, and maintaining them, and (6) patient safety. 34 General surgeons should utilize these.

The practice of surgery is changing in significant ways, and for the good. Novel techniques often require an entirely novel skill set for surgeons. General surgeons should embrace and master new techniques throughout their careers.

The latest trend in training in the context of a larger curriculum of proficiency-based training in surgery is encouraging. It is the best way to achieve good training results. 35 Procedural skills training in a simulated environment, including virtual reality training, has been shown to transfer to the real-life clinical setting. Virtual surgical planning increases confidence and knowledge for surgery for better outcomes. 34 All this should be utilized for training many, for serving all.

## Conclusion

“Vayam Sevaamahe – We are for service” is the motto of the Association of Surgeons of India. Surgical care and cure for all should not be a distant dream in India and worldwide, with dedication and devotion of all the general surgeons. General surgeons should strive for service improvement and innovations in delivery of services for the benefit of patient care. The field of surgery is an ever-evolving one. General surgeons should improvise ways for delivery of all advances for the benefit of all.

In the present context the historic Flexner report, 36 which reformed medical education in the United States, should continue to guide, reinforce, and rejuvenate the services by the general surgeons:

“ *Professional patriotism amongst medical men: the regard for the honor of the profession and the sense of responsibility for its efficiency* .”
